# Diaphragm Disease of the Small Bowel Presenting With Intussusception

**DOI:** 10.7759/cureus.20855

**Published:** 2021-12-31

**Authors:** Vanessa E Al-Feghali, Kevin Sigley, Raymond Laird

**Affiliations:** 1 General Surgery, Beaumont Health, Dearborn, USA; 2 General Surgery, Beaumont Health, Trenton, USA

**Keywords:** nsaid abuse, chronic ulcerations, enterography and endoscopy, obstruction, intussusception, strictures, small bowel, diaphragm disease

## Abstract

Diaphragm disease of the small bowel is an uncommon condition with nonspecific symptoms, which causes strictures of the small bowel associated with non-steroidal anti-inflammatory drug (NSAID) use. Due to the nature of the disease process and the strictures it can form, patients often present with a clinical picture suggestive of small bowel obstruction, and the true diagnosis is not confirmed until histopathological examination.

In this article, we present the case of a 73-year-old female with chronic NSAID use and gastrointestinal complaints who had undergone multiple endoscopic procedures which failed to identify the cause of her symptoms. Further investigation with video capsule endoscopy and CT enterography led to a diagnosis of intussusception believed to be caused by a small bowel mass. Retention of the video capsule endoscope prompted the decision to undertake diagnostic laparoscopy with push endoscopy and direct visualization of a string of small bowel strictures in the area of intussusception. This characteristic appearance of the bowel was then confirmed by pathology as diaphragm disease lesions. Diagnosis of this disorder is difficult due to its rarity and common symptoms that make other disease processes seem more probable. Early diagnosis can prompt counseling on cessation of NSAID use and interventions to decrease the risk of complications that may require surgical intervention. Physicians should be able to recognize diaphragm disease of the small bowel as a differential in patients presenting with obstructive bowel symptoms and even rare cases of intussusception in the setting of chronic NSAID use.

## Introduction

The use of non-steroidal anti-inflammatory drugs (NSAIDs) is very common among most age groups, especially in those with chronic pain and rheumatological disorders. NSAIDs have been known to cause adverse effects on many organ systems including the gastrointestinal tract, renal system, and cardiovascular system. Diaphragm disease of the small bowel is a rare disorder caused by chronic NSAID use and the reactive process it has on the intestinal lumen. Strictures can form throughout the small bowel, causing narrowing and occasionally resulting in bowel obstruction. Given the rarity of the disease as the cause for small bowel obstruction, diaphragm disease is difficult to diagnose without histopathological analysis. Physicians who are familiar with the symptoms of this disease and its potential complications are at an advantage for correctly identifying the disorder and performing the necessary steps for treatment.

This article was previously presented as a poster at the 2021 Society of American Gastrointestinal and Endoscopic Surgeons annual meeting on August 31, 2021.

## Case presentation

Diaphragm disease is a rare cause of small bowel obstruction caused by the use of NSAIDs resulting in stricture formation. In this article, we present a case of a 73-year-old female with a history of anemia and chronic back pain for which she used NSAIDs frequently, who complained of nausea, vomiting, abdominal pain, diarrhea, and weight loss. She was initially diagnosed with collagenous colitis by pathological analysis of biopsy samples and underwent multiple endoscopies over several years.

On esophagogastroduodenoscopy (EGD), she was found to have mid-esophageal, pyloric, and duodenal strictures with ulcerations, which were treated with endoscopic dilation. A recent colonoscopy revealed an ascending colon ulcer and chronic active ileitis concerning Crohn’s disease; however, this could not be confirmed by pathology. She was maintained on steroids and was trialed on medications for the treatment of possible Crohn’s disease. Against recommendations, she continued to take NSAIDs, and her symptoms persisted. She then underwent video capsule endoscopy with a PillCam™ (Medtronic, Minneapolis, MN), which demonstrated numerous small bowel ulcers and inflammation consistent with Crohn’s disease; however, the PillCam™ did not reach the colon. After nearly a month without evacuation of the PillCam™, CT enterography was performed and revealed intussusception with a possible small bowel mass. She continued to have abdominal pain, nausea, and vomiting over the next few months. Despite passing bowel movements and lack of complete bowel obstruction, she did not pass the PillCam™.

**Figure 1:**
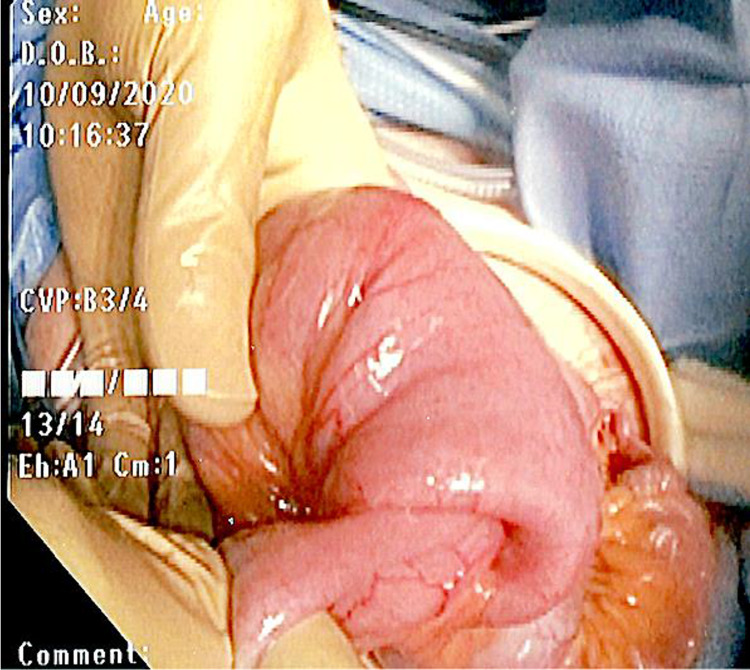
Intraoperative photo of intussusception

**Figure 2:**
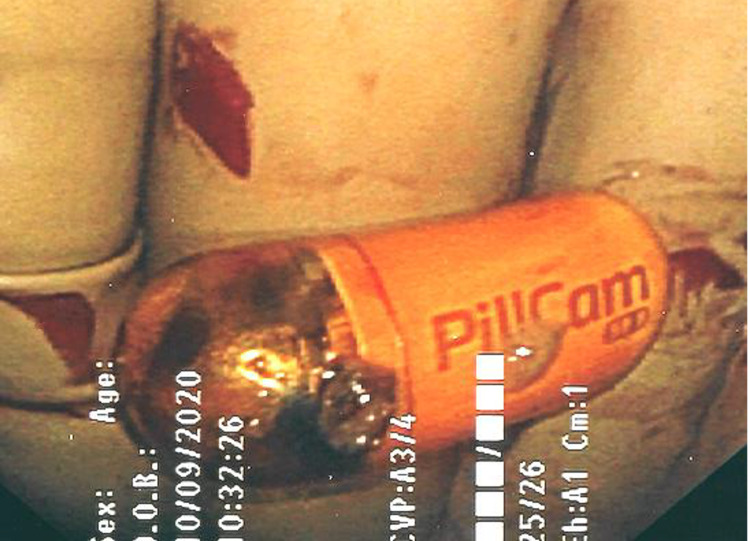
Video capsule endoscope retrieved intraoperatively

**Figure 3:**
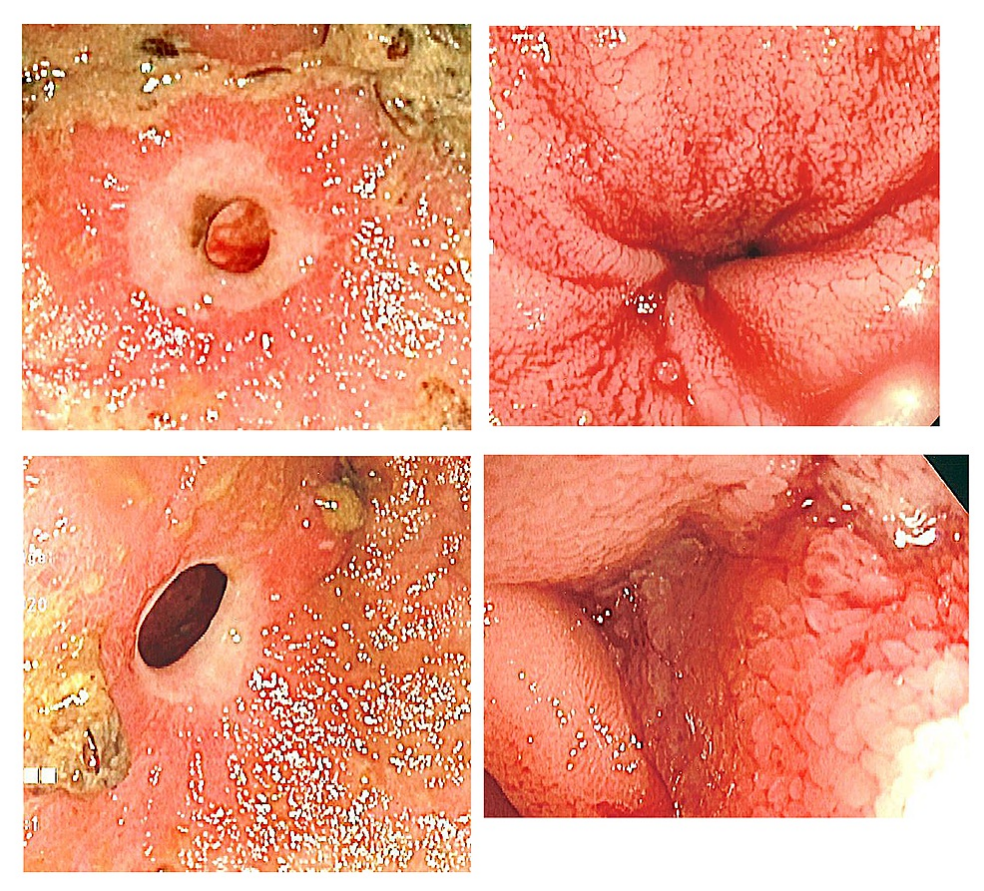
Endoscopic photos of small bowel diaphragm stricture

The decision was made to proceed with diagnostic laparoscopy with collaboration with her gastroenterologist to perform intraoperative push endoscopy through planned enterotomies. During the diagnostic laparoscopy, a wound protector was used to extracorporealize the bowel (Figure 1). Enterotomies were made, and push endoscopy was performed until the area of intussusception and PillCam™ was palpated and visualized. The intussusceptum appeared to encompass a 10-cm mass. Another enterotomy was made distal to the mass, and push endoscopy was continued to the cecum with the removal of the PillCam™ and one polyp (Figure 2). A total of approximately 14 strictures were visualized, both proximal and distal to the mass (Figure 3).

The small bowel ileum was resected to include the entirety of the strictures, the area of intussusception, and the presumed mass. A functional side-to-side anastomosis was created, and the patient did well in the postoperative period. The specimen was analyzed by pathology and measured approximately 39 cm in length. It was identified as a large inflammatory small bowel fibroid polyp with associated mucosa ulceration, serositis, and serosal adhesions consistent with intestinal intussusception and multiple diaphragm disease lesions. The area of intussusception measured 6.0 cm x 5.5 cm x 4.5 cm, and there were four circumferential fibrous strictures situated on either side of the mass, causing the lumen to narrow to a diameter of 1 cm. There was no evidence of dysplasia or malignancy. The patient was educated on cessation of NSAID use to prevent recurrence of stricture formation and her initial presenting symptoms.

## Discussion

Small bowel diaphragm disease is a rare cause of small bowel obstruction that is often misdiagnosed due to the rarity of the condition and its presenting symptoms. The appearance of the small bowel lumen as concentric rings of fibrotic tissue defines the term "diaphragm" disease [1,2]. Stricturing of the small bowel is commonly seen in Crohn’s disease due to the recurrent episodes of inflammation and fibrosis. Other less common causes include ingestion of potassium chloride tablets, radiation exposure, anastomotic sites, tuberculosis, small and large bowel lymphomas, and low flow states [3]. Thus, the diagnosis of diaphragm disease requires the exclusion of other common causes of stricture formation.

Risk factors for diaphragm disease of the small bowel include female gender, advanced age, and prolonged NSAID use, oftentimes in those with chronic back pain and arthritis [1,4,5]. Many have average use of NSAIDs for at least three to five years, though as little as two months of NSAID usage has been shown to cause these strictures [4,6]. Of chronic NSAID users, it is suspected that about 2% will develop small bowel diaphragm disease [1,7,8]. Presenting symptoms include chronic abdominal pain, iron deficiency anemia, weight loss, and constipation. If symptoms develop more acutely, imaging often shows findings concerning adhesions or a mass causing small bowel obstruction [5]. Intussusception has not been explicitly discussed in the literature, as in this unique case, but it can be an identifiable presentation in patients with small bowel disease of the diaphragm. It is known that intussusception transpires when one part of the bowel telescopes itself into another segment of the bowel. Intussusception can occur in adults throughout the gastrointestinal tract and is often caused by tumors, adhesions, or other noninfectious or infectious disease states and the impact they have on bowel activity. In this case, it can be hypothesized that the strictures altered the normal peristalsis of the intestinal lumen, forming an area of intussusception that caused a partial bowel obstruction and unusual presentation. Consequently, these strictures can be misdiagnosed both preoperatively and intraoperatively due to the rarity and unfamiliarity of the disease. Without its consideration as a differential diagnosis, physicians may not make the appropriate decisions in treatment and future counseling.

Small bowel strictures were pathognomonic of Crohn’s disease until several decades ago when microscopic analysis of resected bowel facilitated the delineation of diaphragm disease. The effect of chronic NSAID use on the small bowel was first discovered histologically in 1988 [1,5]. Characteristic findings include thickening of the muscularis mucosa, fibrous change to the lamina propria, eosinophilic mucosa, and ulceration of the diaphragm [3]. It is believed that NSAIDs cause increased susceptibility of the small bowel mucosa to the inflammatory responses to bacteria and digestive enzymes by inhibiting prostaglandins and hindering the intestinal villous microcirculation [2,5,7,8]. This results in inflammatory circumferential injury to the submucosa, forming ulcerations that scar and stricture the lumen when healed [5,8]. It is the erosive nature of the disease and the concomitant ulcers that can form causing the characteristic chronic anemia seen in patients [6,8]. This repetitive process can then ultimately form focal areas of stricture [5].

Often the terminal ileum is spared, with an abundance of eosinophils seen on microscopic examination [5]. Due to the hypothesis of prostaglandin inhibition being a factor in the pathological process of the disease, supplementation with prostaglandin derivatives in patients who are unable to stop taking NSAIDs has been suggested [6]. Some propose that diaphragm disease is better correlated to the use of traditional NSAIDs over selective COX-2 inhibitors [6]. Diclofenac has been found to be one of the most frequently used NSAIDs by patients diagnosed with this disorder [9]. Diaphragm disease can be distinguished histologically from inflammatory bowel disease by its typical limitations to the mucosa and submucosa, without full-thickness disruption [6]. It can cause thickening of the muscularis mucosa without penetrating the serosa [6,10]. Although diaphragm disease is more known in the small bowel, there have been reports of colonic involvement: ascending colon more than transverse or descending [6,9]. The presentation of the colonic disease is similar to that of those affected by small bowel disease, with anemia and bowel obstruction, and the findings on histology showing ulceration and fibrosis mimicking those seen in stricturing of the small bowel [9]. It is hypothesized that NSAIDs with longer half-lives that have not been fully metabolized upon reaching the colon have this impact [6,9]. The risks and benefits of NSAID use need to be truly assessed due to the negative effects it can have throughout the gastrointestinal tract.

Diaphragm disease of the small bowel cannot be easily identified due to the nature of the disorder and the way it impacts the gastrointestinal tract. Although it is typically diagnosed on histopathological analysis, there are some imaging studies that can guide physicians to the correct etiology. Balloon-assisted enteroscopy and video capsule endoscopy are the best forms of optical imaging to diagnose diaphragm disease without surgery [11]. CT and MRI enterography can show nonspecific strictures, similar in appearance to those in Crohn’s disease and radiation-induced enteritis [11]. MRI enterography can provide better evaluation and assessment for small bowel strictures than CT imaging.

If the diagnosis is made early, surgery can sometimes be avoided, and patients can be treated with endoscopic balloon dilatation of the small bowel strictures, in addition to cessation of NSAID use [1,7]. When diagnostic laparoscopy is pursued, it can still fail to diagnose this disease as the external appearance of the small bowel may appear grossly normal due to the lack of transmural involvement [3,7]. It is common to find proximally dilated small bowel with distal narrowing, which can be characteristic of several different disease processes. Laparotomy facilitates palpation and tactile appreciation of the strictures and thickening of the intestinal lumen [7,10]. There can be as few as one diseased segment, though it is more common to find multiple, short segments of stricturing [6,10]. The mass-like manifestation of the disorder is often assumed to be a tumor until histopathological analysis [2,7]. Intraoperative enteroscopy is recommended if diaphragm disease is suspected as it allows for intraluminal assessment of further diseased segments of the small bowel that should be resected to prevent recurrence of symptoms [7].

With enteroscopy, the degree of luminal narrowing can range greatly from mild luminal disparity to the near-total obliteration of the luminal opening, causing a mechanical obstruction [5,6]. If this is not performed, capsule endoscopy can be considered after a negative diagnostic laparoscopy [2,7]. The capsule may never be evacuated, prompting the diagnosis but necessitating surgical intervention for retrieval and symptom relief as retained capsules have been shown to cause small bowel obstruction [7]. Stricturoplasty of the affected areas of the small bowel is often undertaken due to misdiagnosis or when the extent of small bowel involvement makes resection unfavorable [3]. There are some studies that have tried to investigate the success of double-balloon enteroscopy with dilatation in diaphragm disease cases [7]. One study involved only a few patients and lacked long-term follow-up [7]. There have been larger studies illustrating the effectiveness of double-balloon enteroscopy in other disease states with known small bowel strictures [12]. One systematic review found that patients who underwent dilatation for small bowel strictures of mixed etiology were able to avoid surgery 80% of the time in an average 2.5-year follow-up [12]. 

Yet, there remains limited data in regard to the efficacy of dilatation and recurrence rates in diaphragm disease. Resection remains the optimal treatment in most situations as it is the most definitive management to prevent recurrence and confirm the diagnosis. Nonetheless, if there is a high index of suspicion for diaphragm disease of the small bowel based on imaging and patient history, resection may not be necessary. 

## Conclusions

Histopathological analysis of the intestinal lumen of the small bowel is often necessary to correctly diagnose diaphragm disease. Chronic NSAID use appears to cause the stricturing that is often missed on diagnostic laparoscopy but palpated on laparotomy and visualized during enteroscopy. The presenting symptoms are nonspecific for diaphragm disease but relate to the partial or intermittent small bowel obstruction caused by these strictures. Surgical resection is often undertaken when patients present acutely with obstructive symptoms; nonetheless, stricturoplasty and balloon dilatation have been shown to be effective treatment options when combined with NSAID cessation. This case demonstrates that it is imperative that physicians be mindful of this disorder and the wide range of effects that NSAIDs can have on the bowel to avoid unnecessary surgery in patients with diaphragm disease.
